# Pediatric inguinal-perineal cavernous hemangioma: A rare presentation and surgical outcome

**DOI:** 10.1016/j.ijscr.2025.111100

**Published:** 2025-02-28

**Authors:** Qasem N. Dola, Muhammad Takhman, Moath Hattab, Mustafa A. Shahrori, Saed Bani Amer

**Affiliations:** a Jaffa Specialized Surgical Hospital, Palestine; b Faculty of Medicine and Health Sciences, An-Najah National University, Nablus, Palestine; c Department of Medicine, Faculty of Medicine and Health Sciences, An-Najah National University, Nablus, Palestine; d Princess Basma Teaching Hospital, Jordan

**Keywords:** Hemangiomas, Vascular tumor, Inguinal swelling, Perineal extension, Pediatric case

## Abstract

**Introduction:**

• Infantile hemangiomas are common vascular tumors that often regress spontaneously. However, complications such as disfigurement, functional impairment, or ulceration may require intervention. Imaging modalities, including Doppler ultrasound and MRI, are crucial for identifying vascular involvement and guiding management decisions.

**Case presentation:**

• A 7-year-old male presented with a painful, oval-shaped swelling in the right inguinal region, measuring approximately 4 × 2 cm and extending toward the perineal area. The lesion was firm, smooth, erythematous, and well-defined, with no inguinal lymphadenopathy or cough impulse. Despite initial treatment with oral antibiotics for presumed inguinal lymphadenitis, the mass progressively enlarged. Investigations, including routine blood tests, were normal. Imaging revealed a multilocular, cystic lesion with vascular involvement. Surgical excision of a 5 × 2 × 1.5 cm hemorrhagic, multilocular lesion was performed, and histopathology confirmed the diagnosis of hemangioma.

**Discussion:**

• This case highlights the importance of imaging and multidisciplinary management in diagnosing and treating vascular lesions with atypical presentations. Although most infantile hemangiomas are self-limiting, surgical intervention is warranted for progressive lesions causing discomfort or posing a risk of complications. Prompt surgical excision alleviates symptoms and prevents functional and cosmetic consequences, emphasizing individualized patient care.

**Conclusion:**

• This case underscores the need to consider hemangiomas in the differential diagnosis of atypical pediatric inguinal masses. Early imaging and multidisciplinary management are critical for accurate diagnosis and effective treatment. Surgical excision is a definitive option for symptomatic or progressive lesions, ensuring symptom relief and preventing complications.

## Highlights

• Imaging confirmed a multilocular lesion with vascular and hemorrhagic features.
• Progressive swelling required surgical excision for definitive treatment.
• Surgery revealed a hemorrhagic lesion causing discomfort and risk of complications.
• Multidisciplinary care addressed diagnostic uncertainty and surgical planning.
• Early surgical intervention prevents functional and cosmetic complications.

## 1 Introduction

Inguinal angioma is a relatively rare, localized, benign vascular aberration defined by disproportionate development or expansion of blood vessels in the inguinal regions. These vascular lesions can vary in size and might involve capillaries, veins, or either. As a result, it can have varying appearances: it may seem bright red when composed of small blood vessels (capillary angioma), bluish for larger, dilated vessel walls (cavernous angiomas), or purple for deeper veins (venous angioma) [[1], [2], [3]].

The inguinal region is anatomically complicated and frequently exposed to mechanical friction, which can contribute to the formation or aggravation of vascular abnormalities. Hormonal factors, localized inflammation, and trauma may together contribute to the formation or progression of angiomas [3,4].

While most angiomas are asymptomatic, those in the inguinal region can be unbearable, particularly when scraping against clothing or moving around. Hemangiomas are believed to cause two essential complications: compression and bleeding. Compression occurs when the tumor invades surrounding structures, causing crucial organs, nerves, or tissues to malfunction or appear dislodged. This is particularly hazardous when hemangiomas form near the airway, gastrointestinal tract, or spinal cord. Hemangiomas are very vascular, which can lead to bleeding. These tumors are composed of atypical blood vessels, rendering them prone to rupture. This can cause bleeding, which can occur spontaneously or because of trauma or external pressure. These concerns emphasize the significance of early detection, monitoring, and intervention to avoid serious outcomes. Other concerns may include infection, thrombosis, and ulceration. The expansion of the hemangioma might compress surrounding nerves and blood vessels, resulting in discomfort and functional impairment. Its delicate blood arteries render it susceptible to rupture, resulting in spontaneous bleeding, and trauma can raise the risk of infection and ulceration. Thrombosis can also develop when clots form in dilated arteries. Furthermore, the size and location of the hemangioma might pose esthetic issues and limit movement. The diagnosis is merely clinical, depending on visual examination and palpation. Dermatoscopy may assist in distinguishing angiomas from other vascular or pigmented lesions. In more complex or unique situations, imaging procedures such as Doppler ultrasound or magnetic resonance imaging (MRI) may disclose additional details concerning vascular involvement [[4], [5], [6]].

Most infantile hemangiomas develop naturally before hampering spontaneously. Therefore, simple cases require only monitoring. If a hemangioma affects crucial functions, produces considerable disfigurement, or leads to consequences such as ulceration, aggressive treatment is required. Oral propranolol, a beta-blocker widely known for its efficiency and safety in facilitating lesional regression, is frequently used as the first-line therapy. Alternative therapy, such as topical beta-blockers, systemic corticosteroids, or surgical alternatives, may be considered in circumstances where treatment is ineffective or complex [7,8].

## 2 Case presentation

A 7-year-old male was brought in by his family after they observed a painful, oval-shaped enlargement in the right inguinal area that was approximately 4 × 2 cm. The enlargement propagates into the perineal area, close to the right hemiscrotum, but lacks connection to the scrotum or the right testicle. The surrounding layer of skin is erythematous, and the swelling is attentive to the fingertips. It is harsh, with a smooth surface and well-defined edges, nevertheless not flared to the touch. There is no cough impulse, and no inguinal lymphadenopathy was observed.

The patient was a product of a full-term, normal vaginal delivery, without postnatal complications or NICU admission. He endured a right inguinal herniotomy at the age of 2 years. Initially, the condition was inguinal lymphadenitis, thus oral Augmentin was offered. However, the expansion failed to diminish and became gradually larger.

**Fig. 1:**
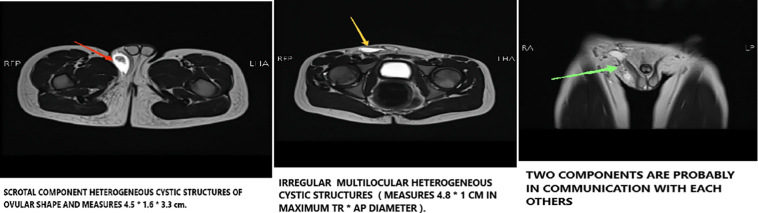
MRI showing oval-shaped and multilocular lesion, measuring 4.5 × 1.6 × 3.3 cm (CC, TR, and AP dimensions, respectively).

**Fig. 2:**
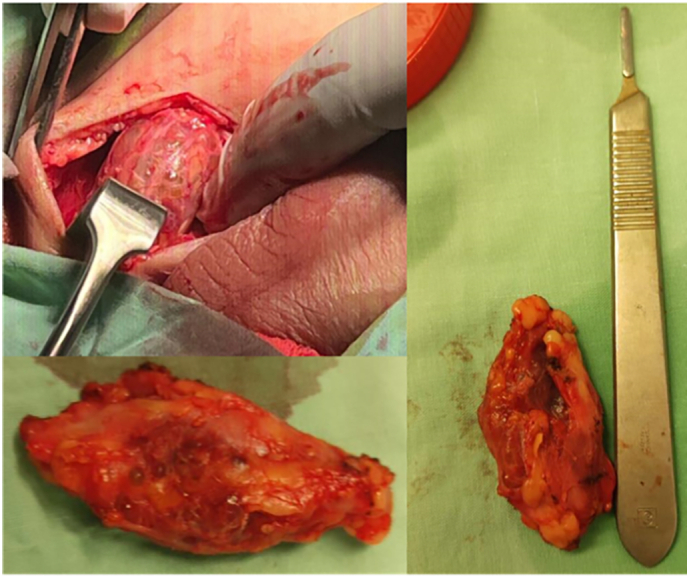
The mass measuring 5 × 2 × 1.5 cm with hemorrhage that was excised.

Investigations, involving CBC, KFTs, LFTs, ESR, LDH, and CRP, were carried out, and all were within normal reference limits. An ultrasound demonstrated a well-defined, homogenous hyperechoic mass with a cystic component. An MRI was also done, which revealed a heterogeneous, cystic lesion in the subcutaneous tissue of the right inguinal region, reaching the scrotal level, and placed posterolateral to the right inguinal canal. The lesion was oval-shaped and multilocular, measuring 4.5 × 1.6 × 3.3 cm (CC, TR, and AP dimensions, respectively) (Fig. 1). Vascular components were identified among the swelling. The differential diagnoses included hemangioma, angiolipoma, and fibro-angioma. Surgically excised a red/grayish lesion measuring 5 × 2 × 1.5 cm with cavities filled with hemorrhagic fluid (Fig. 2).

**Fig. 3:**
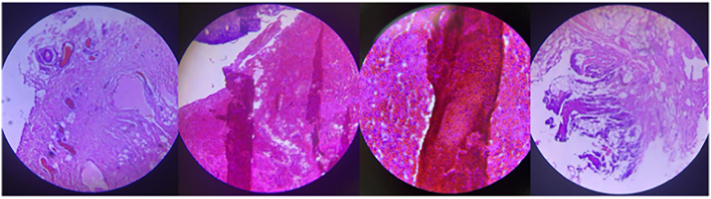
Step sectioning of the mass revealed cavities filled with hemorrhagic fluid, and another fragment of fibrofatty tissue, consistent with hemangioma with degeneration and reactive inflamation.

A biopsy was sent over for histological examination, which revealed cavities filled with hemorrhagic fluid and another fragment of fibrofatty tissue, consistent with the diagnosis of hemangioma with degeneration and reactive inflammation (Fig. 3).

The patient was followed up after one week, one month, and six months, with no evidence of recurrence. The patient's family has been advised to keep an eye on other potential swellings.

## 3 Discussion

One of the most widespread congenital abnormalities is hemangioma, a benign vascular tumor characterized by the proliferation of endothelial cells. It is believed to mostly affect the liver, subcutaneous tissue, and skin. Hemangiomas, or genuine vascular tumors, are categorized as benign tumors by the International Society for the Study of Vascular Anomalies (ISSVA) since result from endothelial cell hyperplasia. Conversely, vascular malformations are classified according to blood flow characteristics and include venous, lymphatic, and arteriovenous malformations. Also, believed to be local deviations in morphogenesis with endothelium with inappropriate cell proliferation [9].

Gibson classifies scrotal vascular lesions into two categories: hemangioma of skin, which originates from the scrotum's dermis, and hemangioma of sub-conductive tissue, which develops from the scrotum's subcutaneous tissue. The former, also known as angiokeratoma, is very prevalent in older adults [10]. Intra-scrotal hemangioma, which corresponds to the latter, is rare with less than 50 cases published [11]. In more recent year immunohistochemical studies can be used to distinguish the development stages of hemangiomas for example endothelial cells express CD31, von Willebrand factor, and urokinase in all phases while infantile hemangiomas express glucose transporter protein-1 (GLUT 1) during all phases of their development [12].

Cavernous hemangiomas of the genitourinary tract may develop almost anywhere, including the glans, penile shaft, scrotum, and perineum, and may even be present from birth. Typically, these lesions appear during adolescence as painless penoscrotal masses, even though they may occasionally result in discomfort, ulceration, or bleeding. Cavernous hemangiomas, unlike other vascular lesions, may not involute with time but rather expand with growth, occasionally reaching into the perineum, anterior abdominal wall, or pelvis. These lesions may have a blueish hue and a rugose or bosselated skin appearance. Hemangiomas tend to be sporadic, nevertheless, they might be related to other disorders like Fabry disease or Klippel-Trenaunay Syndrome Hemangiomas can appear equitably on either side of the scrotum, sometimes resembling a right varicocele. These vascular lesions can exert local pressure, causing discomfort, and may pose risks of hemorrhage, infection, and hematuria, particularly when associated with bladder hemangiomas, or effects on spermatogenesis, notably in deep scrotal hemangiomas that extend to surrounding pelvic structures [10,11,13].

Lesions that propagate from the scrotum to surrounding locations, such as the perineum, thigh, or abdominal wall, are barely documented and can be difficult to diagnose and treat. In circumstances like this condition, a subcutaneous scrotal-perineal hemangioma might mimic an inguinal hernia [10].

Although ulceration is the most prominent outcome, fertility remains a major concern to worry about, since reports imply potential impacts induced by the elevated temperature created by the hemangioma scrotal hemorrhage following trauma, or smaller testicles on the same side as the hemangioma. Two incidents of azoospermia have been documented, presumed to be caused by an increase in temperature in the scrotum [11,[14], [15], [16]].

Other documented significant consequences include testicular torsion [17] and testicular infarction [18]. Ultrasound imaging endures to be a significant diagnostic tool for identifying unique characteristics, such as hypoechoic regions with hyperechoic findings, which facilitates more accurate diagnosis and treatment planning for these scarce but potentially serious lesions [19]. Although color Doppler may indicate blood flow inside these lesions, the lack of flow does not rule out the existence of these lesions [20]. Other imaging modalities, such as magnetic resonance imaging (MRI), are valuable for determining the presence and extent of hemangiomas, particularly when analyzing deep- seated or confusing vascular lesions since they give precise information on vascular anatomy and flow dynamics [20]. Historically, benign hemangiomas were frequently surgically removed due to worries about traumatic rupture and extensive bleeding, even though these scenarios are infrequent. Additional considerations, like cancer risk, persistent discomfort, reproductive concerns, and cosmetic outcomes, continue to require surgical intervention in many cases, particularly with scrotal hemangiomas. In these circumstances, deliberate excision is generally indicated to preserve the scrotum's anatomical integrity. For endoscrotal hemangiomas, precise enucleation that minimizes testicular tissue removal is typically advised [10].

The decision for surgical intervention in this case was based on several key factors. Despite conservative treatment, the mass continued to enlarge progressively. Additionally, the presence of pain and erythema indicated the potential for complications. The mass's location in the inguinal and perineal regions, along with imaging findings showing vascular components and cystic degeneration, further supported the need for surgical removal. The patient did not have significant risk factors such as a genetic predisposition or underlying systemic conditions, which could have complicated the case further. This intervention was recommended to prevent further progression and alleviate symptoms.”

Due to its vasoconstrictive, anti-angiogenic, and apoptotic effects, oral propranolol is now chosen as the initial regimen for infantile hemangiomas. Propranolol is most effective during the development phase of the lesion, thus early identification and therapy are critical—ideally within the first six months. Propranolol's efficacy decreases if it is initiated later than this. When propranolol is ineffective, surgical alternatives remain, intending to minimize tissue loss while preserving function [11]. Various therapies, such as vascular ligation, steroid injections, orchiectomy, and radiation, have been sought throughout the years, but they have mostly been abandoned owing to unsatisfactory outcomes [10].

For smaller, superficial scrotal hemangiomas, less invasive methods such as pulsed dye laser therapy, cryotherapy, and sclerotherapy with agents like polidocanol or hypertonic saline seem promising, particularly for tiny lesions with a high cosmetic priority. Nd: YAG and CO2 laser treatments are adequate for small glandular lesions, although both are expensive and have the potential to result in scarring. When administering significant doses of sclerosing drugs, take precautions to avoid consequences such as tissue necrosis, thrombophlebitis, and embolism [13].

For larger or more complex hemangiomas, where conservative approaches may be insufficient, surgical removal is frequently the preferred option to guarantee total excision in one session [13]. In some scrotal tumors, en bloc resection, which involves removing both the lesion and surrounding tissue, may be required to limit the prospect of retaining tissue (rare) and complications such as disseminated coagulation problems, especially in cavernous hemangiomas [10]. Postoperative healing times vary depending on the size and intricacy of the lesion, as well as the patient's overall condition [16].

Selective embolization, a minimally invasive treatment, is another valid treatment option as a primary treatment in inoperable cases or preoperatively to shrink the tumor size and prevent bleeding. Inability to catheterize and low blood flow of the feeding vessels are serious limiting factors for this technique. Recurrence and re-recurrence of pain, ischemic pain due to necrosis and post-embolization syndrome are most common reported side effect [21].

Ultimately, treatment planning should consider lesion location, size, cosmetic concerns, infertility risk, and cost. Given the cosmetic boundaries of surgery, conservative therapy and monitoring may be more appropriate for glans lesions. Regardless of the therapy options, thorough follow-up is essential to monitor outcomes and address potential complications effectively.

## 4 Conclusion

This case highlights a rare presentation of a subcutaneous cavernous hemangioma in the right inguinal and perineal region of a 7-year-old male, which was misdiagnosed initially as inguinal lymphadenitis. The lesion's unique characteristics, including its location, painful nature, and propagation toward the perineum without involvement of the scrotum or testicle, underscore the diagnostic challenge posed by these vascular anomalies.

Surgical excision remains the gold standard for managing such lesions, particularly when conservative measures fail or when there is a risk of complications such as hemorrhage, infection, or functional impairment. In this case, complete excision resulted in resolution of symptoms with no recurrence over a six-month follow-up period.

This case underscores the importance of considering vascular lesions in the differential diagnosis of inguinal and perineal masses in children, particularly when initial treatment fails. Early imaging and histological confirmation are crucial for accurate diagnosis and timely intervention. However, the underlying mechanisms contributing to the progression, degeneration, and complications of hemangiomas remain poorly understood. Further research into the pathophysiology of these vascular anomalies is needed to refine treatment strategies, optimize patient outcomes, and potentially identify novel non-surgical therapeutic approaches. Given the potential for complications, including infertility, testicular damage, or cosmetic concerns, long-term monitoring and patient education are essential.

## Abbreviations

MRI: Magnetic Resonance Imaging
NICU: Neonatal Intensive Care Unit
CBC: Complete Blood Count
KFTs: Kidney Function Tests
LFTs: Liver Function Tests
ESR: Erythrocyte Sedimentation Rate
LDH: Lactate Dehydrogenase
CRP: C-Reactive Protein
CC: Cephalocaudal (used to describe dimensions)
TR: Transverse (used to describe dimensions)
AP: Anteroposterior (used to describe dimensions)
ISSVA: International Society for the Study of Vascular Anomalies

## CRediT authorship contribution statement

Muhammad Takhman and Moath Hattab: writing the paper discussion.

Mustafa A. Shahrori: writing the paper case presentation and data collection.

Saed Bani Amer: writing the paper introduction.

Qasem N. Dola: data collection, overall supervision.

## Informed consent

Parental consent for minor: Written informed consent was obtained from the patient's parents/legal guarding for publication and any accompanying images. A copy of written consent is available for review by the Editor-in-chief of this journal on request. This case report has been reported in line with the SCARE 2023 criteria [22].

## Ethical approval

IRB is not required for case report - Jaffa specialized surgical hospital.

## Guarantor

Qasem N. Dola.

## Funding statement

N/A.

This research did not receive any specific grant from funding agencies in the public, commercial, or not-for-profit sectors.

## Approval of the research protocol by the Institutional Reviewer Board

Is not required by the institution for case reports.

## Declaration of competing interest

N/A.

The authors declare no conflicts of interest.
